# Precision Covalent Chemistry for Fine-Size Tuning of Sandwiched Nanoparticles between Graphene Nanoplatelets

**DOI:** 10.1021/acsomega.3c04727

**Published:** 2023-10-27

**Authors:** Mustafa K. Bayazit

**Affiliations:** † Sabanci University Nanotechnology Research and Application Center, Tuzla Istanbul 34956, Turkey; ‡ Faculty of Engineering and Natural Science, Sabanci University, Istanbul 34956, Turkey; § Department of Chemical Engineering, University College London, Torrington Place, London WC1E 7JE, U.K.

## Abstract

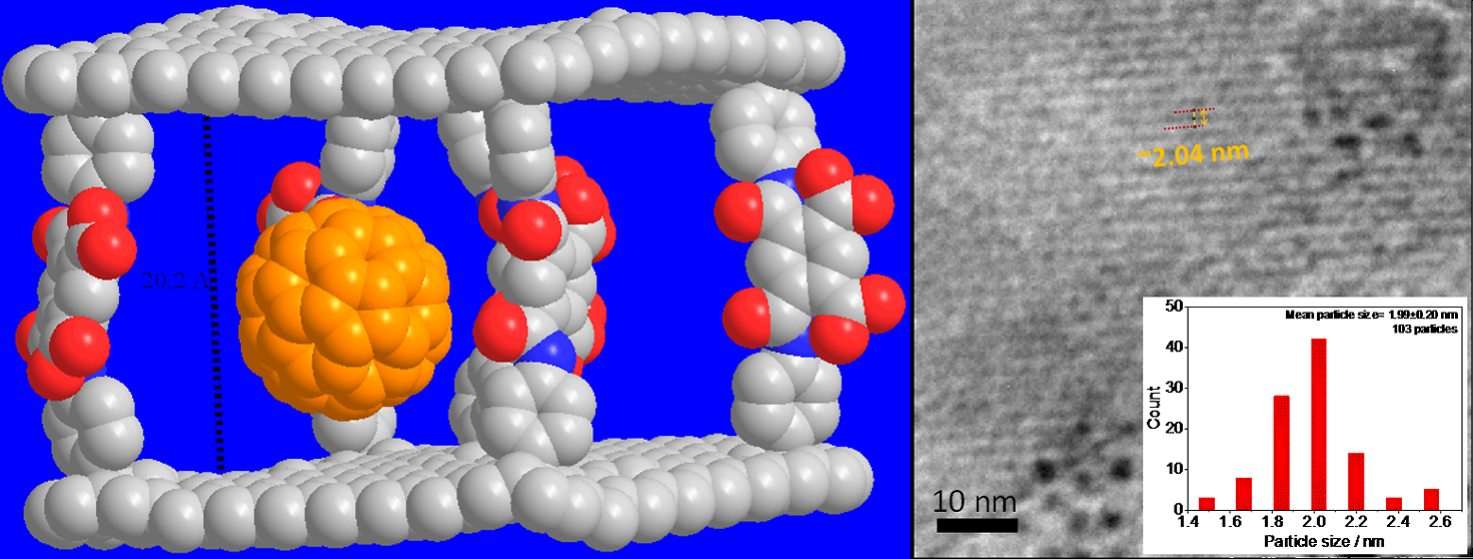

The covalent functionalization of graphene for enhancing their stability, improving their electrical or optical properties, or creating hybrid structures has continued to attract extensive attention; however, a fine control of nanoparticle (NP) size between graphene layers *via* covalent-bridging chemistry has not yet been explored. Herein, precision covalent chemistry-assisted sandwiching of ultrasmall gold nanoparticles (US–AuNP) between graphene layers is described for the first time. Covalently interconnected graphene (CIG) nanoscaffolds with a preadjusted finely tuned graphene layer–layer distance facilitated the formation of sandwiched US–AuNPs (∼1.94 ± 0.20 nm, 422 AuNPs). The elemental composition analysis by X-ray photoelectron spectroscopy displayed an aniline group addition per ∼55 graphene carbon atoms. It provided information on covalent interconnection *via* amidic linkages, while Raman spectroscopy offered evidence of covalent surface functionalization and the number of graphene layers (≤2–3 layers). High-resolution transmission electron microscopy images indicated a layer–layer distance of 2.04 nm, and low-angle X-ray diffraction peaks (2θ at 24.8 and 12.5°) supported a layer–layer distance increase compared to the characteristic (002) reflection (2θ at 26.5°). Combining covalent bridging with NP synthesis may provide precise control over the metal/metal oxide NP size and arrangement between 2D layered materials, unlocking new possibilities for advanced applications in energy storage, electrochemical shielding, and membranes.

## Introduction

Graphene-originated nanostructures are promising for developing advanced applications due to their unique properties, including high conductivity, single-atom thickness, and mechanical flexibility.^1−3^ However, graphene use for high-performance photonic or optoelectronic devices is constrained by its intrinsically low absorption cross-section and quantum efficiency.^4,5^ Thus, graphene/metal nanoparticle hybrid composites, particularly bearing plasmonic nanoparticles [*e.g.*, gold (Au) and silver (Ag)], have gained significant attention to tune the electronic and optical properties of graphene for specific applications.^6−15^

For example, the US–AuNP (2–4 nm)-decorated graphene nanocomposites, prepared by laser irradiation, were reported as promising materials for photothermal therapy and the efficient conversion of solar energy into useable heat for a variety of thermal, thermochemical, and thermomechanical applications.^15^ Zaniewski et al.^16^ sandwiched gold islands (50–200 nm) between the graphene sheets and compared their electronic and optical properties with those of a graphene layer, a gold-decorated graphene layer, and laminated unfilled double layers. The gold-filled sandwiches showed the lowest sheet resistance, a red-shifted broad optical absorption spectrum, and a shifted work function, suggestive of distinct properties from simple graphene laminations or graphene with metallic overlayers. In addition, a concept study that follows graphene growth, nanoparticle decoration, and graphene layer transfer to prepare sandwich structures of graphene (or other 2D sheets) was reported by using various nanoparticles, including Au (∼10–24 nm), Pd (∼15 nm), and PbSe (∼20 nm).^17^ Although this method offered a route for preparing sandwich nanoparticles (≥10 nm) between graphene layers, the size control of nanoparticles was highly dependent on the optimization of reaction parameters, such as precursor concentration and reducing agent. A sacrificed template-based technique was also developed to sandwich large cobalt (Co) nanoparticles and their oxides between carbon nanosheets *via* high-temperature calcination of glucose-based polymer/(NH_4_)_2_Co_8_(CO_3_)(OH)_6_:4H_2_O under an inert atmosphere.^18^ Although ultrasmall nanoparticles (USNPs), with sizes of 1 to 3 nm, are known to have unique properties highly distinct from larger counterparts,^19^ the precision-tuning of NPs’ size between layered nanomaterials has been studied less.

Herein, we report for the first time the synthesis of ultrasmall gold nanoparticles (mean particle size of 1.94 ± 0.20 nm) (US–AuNPs) sandwiched between graphene layers with a preadjusted finely tuned layer–layer distance of ∼2.04 nm.

## Experimental Section

### Materials and Methods

Graphite was provided by Graphexel Limited (graphite grade 2369) and used as received to prepare highly exfoliated graphene flakes following the literature.^20^ Natural graphite flakes [Alfa Aesar, *graphite flake*, *natural*, -*10 mesh*, 99.9% (metal basis)] were used for bulk-functionalization experiments. *N*,*N*-Dimethylformamide (DMF) (ACS reagent, ≥99.8%, Sigma-Aldrich), 1,4-diaminobenzene (≥99.0%, Aldrich), pyromellitic dianhydride (PMDA) (97%, Aldrich), and sodium nitrite (NaNO_2_) (≥97.0%, Aldrich) were purchased from Sigma-Aldrich and used as received. High-purity distilled water was obtained from an Elga PURELAB Prima deionized water machine (15 Ω).

### Preparation of Covalently Interconnected Graphene Flakes with Ultrasmall Gold Nanoparticles

A modified procedure was used to functionalize the surface of the graphene flakes.^21^ In a typical procedure, a cooled solution (0 °C) of NaNO_2_ (0.061 g, 0.884 mmol, dissolved in 0.5 mL of water) was added dropwise to 1,4-diaminobenzene [0.095 g, 0.884 mmol dissolved in 2 mL of hydrochloric acid (HCl)]. The stoichiometric ratio was adjusted to 1 equiv/1 equiv (NaNO_2_/1,4-diaminobenzene) to ensure that only one amine group is diazotized.^22^ The resulting yellow solution was stirred for 30 min and maintained at 0 °C. A cooled solution (0 °C) of highly exfoliated graphene flakes in DMF^20^ (10 mL, ∼15 μg/mL) was then added dropwise. The reaction mixture was held at 0 °C, stirred for 4 h, and then mixed for 15 h at room temperature. The surface-modified graphene flakes were filtered through a PTFE membrane (0.5 μm), redispersed in 2 M HCl (20 mL), and filtered and washed with a copious amount of water until pH was neutral. The resultant solid material was dispersed in 2 M sodium hydroxide (NaOH) (10 mL) and stirred overnight to convert the produced aniline hydrochloride salt to aniline groups. The aniline-modified graphene flakes were then isolated by filtration through a PTFE membrane (0.5 μm), washed with water until pH neutral, redispersed, and filtered using THF (2 × 10 mL), acetone (2 × 10 mL), and ethanol (2 × 10 mL), respectively, and dried at 80 °C in an oven. A bifunctional molecule, PMDA, was used to covalently interconnect aniline-modified graphene flakes. A two-step reaction was followed to ensure the formation of a molecular bridge between the aniline-modified graphene flakes. Initially, the aniline-modified graphene flakes (10 mL) were reacted with excess PMDA in dry DMF at 60 °C to enable only the one-side addition of PMDA to the aniline-modified graphene flakes. Unreacted PMDA was removed by membrane filtration, and the wet cake was washed with DMF (100 mL) and THF (100 mL). The aniline-PMDA-modified graphene flakes were redispersed and filtered using dry DMF (4 × 10 mL) and dry THF (4 × 10 mL), respectively, and then dried under N_2_ flow. The resultant aniline-PMDA-modified graphene flakes were redispersed in dry DMF to prepare a highly stable dispersion. Then, 10 mL of well-dispersed aniline-modified graphene flakes in dry DMF was added to the aniline-PMDA-modified graphene flakes’ solution. The mixture was heated overnight to 60 °C under nitrogen. The above-mentioned purification steps (redispersing, filtration, and solvent washing using DMF and THF) were repeated to isolate the prepared covalently interconnected graphene nanoscaffolds. For bulk functionalization, a graphite dispersion (instead of a stable graphene dispersion) in DMF (10 mg in 20 mL, bath sonication for 30 min) was used to prepare aniline-functionalized graphene nanosheets following the modified literature procedure described above,^21^ and the resulting aniline-functionalized graphene was used for nanoscaffold preparation. AuNPs were prepared in the presence of the covalently linked graphene nanoscaffolds following the Turkevich method.^6,23,24^ In addition, two control experiments were performed with and without aniline-modified graphene. In a typical procedure, ∼10 μg/mL of covalently linked graphene nanoscaffolds was prepared in high-purity water (20 mL) using bath sonication (30 min). Then, 2 mL of freshly prepared aqueous HAuCl_4_ (0.005 M) was added to the covalently linked graphene nanoscaffold solution. This mixture was initially stirred at room temperature for 30 min, and the solution temperature was raised to 90 °C. After that, 1.2 mL of hot (90 °C) Na_3_Cit solution (0.05 M), required to prepare the citrate-to-gold molar ratio of 6-to-1, was added to the HAuCl_4_/covalently linked graphene nanoscaffold solution at 90 °C. The mixture was stirred for an additional 2 h at the same temperature. After cooling to room temperature, it was filtered through a PTFE membrane (0.5 μm) and washed with copious amounts of water to remove the unreacted gold salt precursor. The resultant material was dried at 80 °C in a vacuum oven to give a gold nanoparticle/covalently linked graphene nanoscaffold hybrid structure.

### Materials Characterization

Raman spectra were obtained from a Renishaw InVia Raman Microscope using a 514.5 nm excitation laser and wavenumbers ranging between 100 and 3000 cm^–1^. Purchased graphite flakes and the covalently linked graphene nanoplatelets were tested in powder form. A stable DMF dispersion of the covalently linked graphene nanoplatelets was deposited on the substrate surface and tested in powder form. Raman analysis was performed on a clean Si-wafer substrate to prevent the substrate’s interference with the spectra and to observe any peak shift relative to the Si reference material at 520 cm^–1^. Ultraviolet visible (UV/vis) spectra were obtained from stable dispersions of the produced graphene in DMF using a Shimadzu UV-2550 UV/vis spectrophotometer. High-resolution X-ray photoelectron spectroscopy (XPS) was performed by a Thermo Scientific K-alpha photoelectron spectrometer with monochromatic Al Kα radiation; peak positions were referenced to the C 1s line at 284.5 eV, and the CasaXPS software with Shirley background was used while deconvoluting N 1s spectra. Transmission electron microscopy (TEM) and high-resolution transmission electron microscopy (HRTEM) were performed using Jeol JEM-1010 and JEOL-2010F coupled with EDS detector (Oxford Instruments) instruments, respectively. All TEM samples were prepared in DMF and dropped on carbon-coated copper grids, which were dried in air before imaging. The particle size distribution of AuNPs was estimated from TEM data by using ImageJ software. X-ray diffraction (XRD) analysis was performed using a Bruker D8 ADVANCE 5–90°, 0.02 step size, Cu Kα (1.5406 A) at 40 kV 10 mA, and carbon nanomaterials dispersed in ethanol were used to prepare XRD samples. All graphs, statistical analyses, and Raman spectral fittings were performed using OriginLab software (Origin 9.1).

## Results and Discussion

**Scheme 1:**
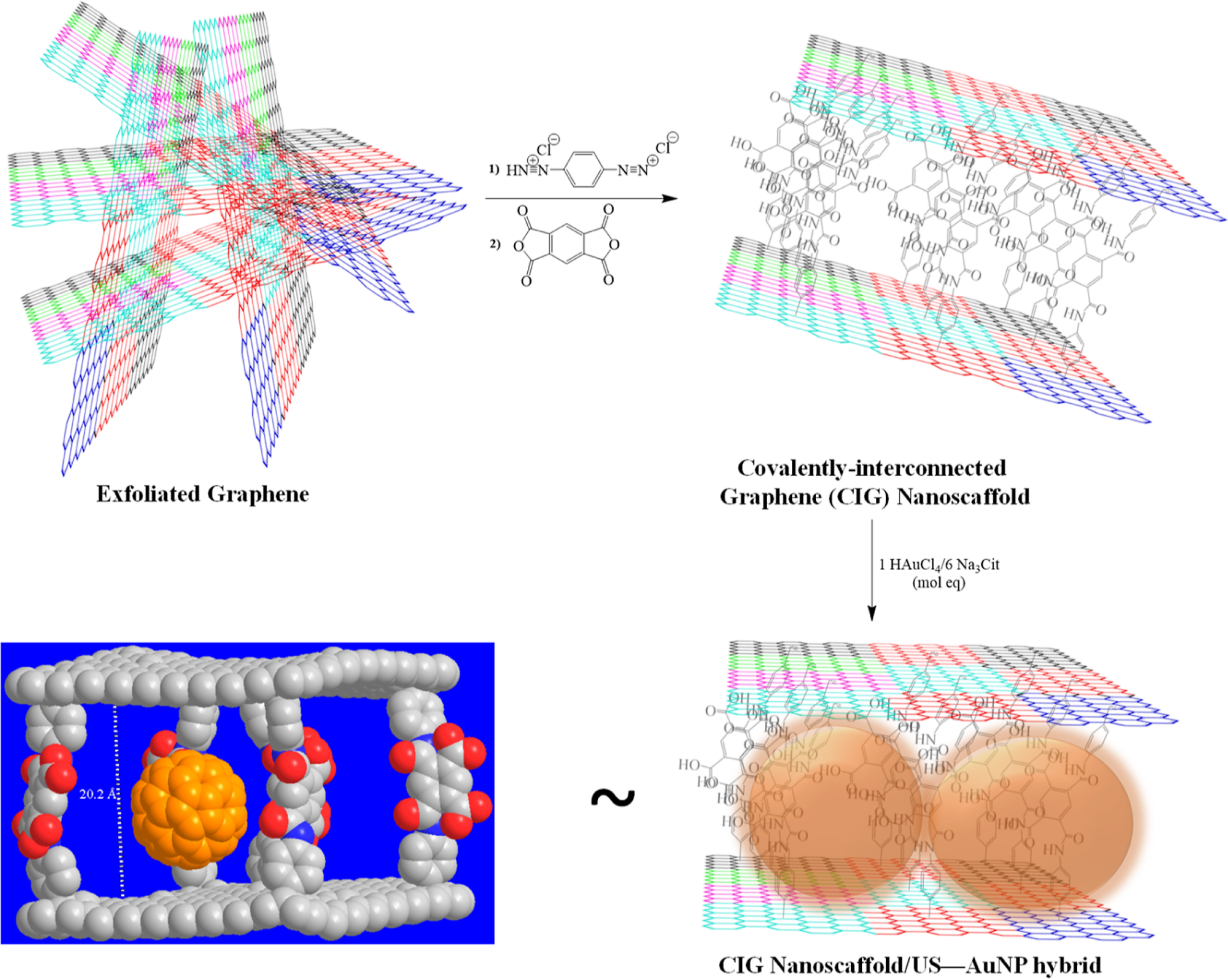
Synthetic Pathway for CIG Nanoscaffolds with a Predefined Layer–Layer Distance and Their Hybrid Structure with AuNPs A simple space-filling model of an ideal CIG nanoscaffold with pyromellitic diamic acid bridges shows the estimated layer–layer distance of ∼2.02 nm. Each graphene layer is formed of ∼200 carbon atoms. Multicolored motifs on individual graphene layers correspond to ∼55 carbon atoms. There is a bridge per every ∼55 graphitic carbon atoms. In the space-filling model, gray, red, and blue atoms refer to carbon, oxygen, and nitrogen atoms, respectively. Orange spheres represent AuNPs grown in between graphene layers.

The molecularly designed CIG nanoscaffolds were prepared *via* chemical reaction-mediated self-assembly of functionalized graphene nanoplatelets. Stable graphene dispersion (∼15 μg/mL) in DMF (see Supporting Information Figure S1 for picture and UV-spectrum of the exfoliated graphene) was used for functionalization experiments, and it was prepared following a literature method,^20^ which produces defect-free single-layer graphene *via* microwave solid exfoliation. In a typical CIG nanoscaffold preparation, exfoliated graphene nanosheets were first functionalized with aniline groups following a modified diazonium salt addition procedure (Scheme 1).^21^ The stoichiometric ratio between sodium nitrite (NaNO_2_) and 1,4-diaminobenzene was 1 equiv to ensure that only a single amine was diazotized.^22^ The aniline-functionalized graphene nanoplatelets were covalently interconnected using a bifunctional linker [pyromellitic dianhydride (PMDA)] to prepare CIG nanoscaffolds.

**Figure 1:**
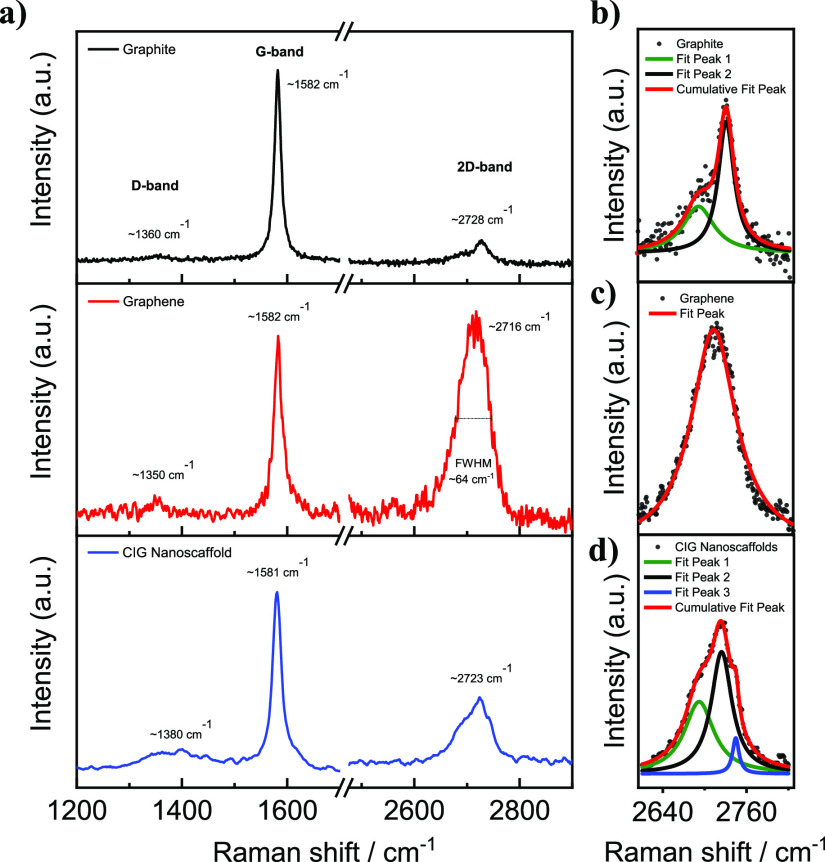
(a) D- and G-band region Raman spectra of pristine graphite, exfoliated graphene, and CIG nanoscaffolds, normalized at the G-band (the D- and G-bands are observed between 1200 and 1700 cm^–1^). The 2D-band region Raman spectra of (b) pristine graphite, (c) exfoliated graphene, and (d) CIG nanoscaffolds. The 2D-band region Raman spectrum of the CIG nanoscaffolds is fitted by a Lorentzian function. The analysis is carried out on a Si-wafer surface by using a laser excitation of 514 nm.

The covalent surface functionalization of graphene nanoplatelets was evaluated by Raman spectroscopy.^25−27^ The D-band observed at *ca.* 1350 cm^–1^ is widely regarded as a reliable measure of surface defects and chemical functionalization. Therefore, the area ratio of the D-band to the G-band (*A*_D_/*A*_G_) is used to determine the degree of surface defects on a graphitic material.^25^ Raman spectra of pristine graphite and CIG nanoscaffolds are shown in Figure 1a–d. The CIG nanoscaffolds exhibit an *A*_D_/*A*_G_ ratio of 0.36, compared to 0.07 and 0.11 for graphite and exfoliated graphene, respectively, indicating the successful covalent addition of aniline groups to the graphene surface (Figure 1a).

The characteristic 2D-band of graphene is attributed to a two-phonon double resonance process, which broadens upon an increased number of graphene layers.^28^ The exfoliated graphene shows an *I*_2D_/*I*_G_ ratio of ∼1.14 at *ca.* 2716 cm^–1^, and the shape, location, and intensity of the 2D-band fitted by a single curve suggest the presence of thin graphene layers (≤2–3 layers) compared to the pristine graphite with an *I*_2D_/*I*_G_ ratio of ∼0.14 at *ca*. 2728 cm^–1^ (Figure 1a–c).^20^ Furthermore, the exfoliated graphene’s broad fwhm (∼64 nm) suggests the formation of rotationally reordered single-layer graphenes.^29^ On the other hand, the 2D-band of the CIG nanoscaffolds displays an intense peak at *ca*. 2723 cm^–1^ (*I*_2D_/*I*_G at 1580 cm_^–1^ = 0.41) (Figure 1a), and an evident change in the shape of the 2D-band of the CIG nanoscaffolds is observed compared to that in the pristine graphene fitted by two peaks. The 2D-band of CIG nanoscaffolds is fitted by three Lorentzian peaks (fit peaks 1, 2, and 3 in Figure 1d), characteristic of four-layer graphene flakes, which is in close agreement with the previously reported four-layer graphene flakes.^29^ Overall, Raman analysis suggests that few-layer graphene architectures are formed due to the chemical reaction-mediated self-assembly of functionalized graphene nanoplatelets *via* PMDA bridges.

The covalent molecular interconnection between the graphene layers was further characterized by X-ray photoelectron spectroscopy (XPS). The aniline-functionalized graphene flakes shown in Scheme 1 were used to prepare CIG nanoscaffolds. It is well known that diamines readily react with dianhydrides to yield poly(amic acid) bearing amide and carboxylic acid functional groups.^30^ In such reactions, primary amine groups convert into amide linkages; thus, one can expect a chemical environmental change in the nitrogen atom of aniline. Furthermore, adding a bulk group such as the oxygen-rich PMDA (C_10_H_2_O_6_) results in a considerable difference in the elemental composition of the functionalized graphene compared with that of the pristine one.

**Figure 2:**
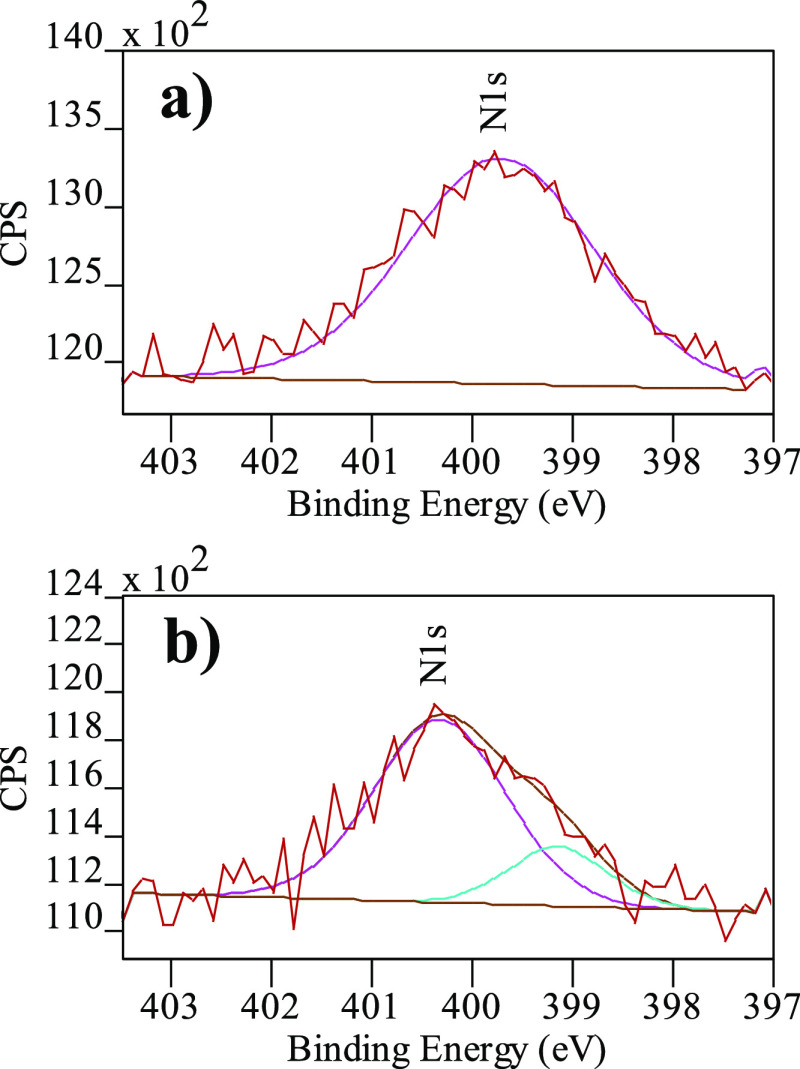
N 1s XPS spectra of (a) aniline-functionalized graphene and (b) CIG nanoscaffolds.

The XPS elemental analysis of the aniline-functionalized graphene nanoplatelets shows 91.68, 6.84, and 1.48 atom % C (284.68 eV), O (532.78 eV), and N (399.78 eV), respectively (see Supporting Information Figure S2 for high-resolution XPS spectra of C 1s and O 1s). The elemental composition of nitrogen (1.48 at %) corresponds to approximately 1 aniline group per 55 graphitic carbon atoms. In addition, the XPS elemental analysis of the CIG nanoscaffolds shows 81.96, 17.32, and 0.72 atom % C (284.68 eV), O (532.28 eV), and N (400.38 eV), respectively (see Supporting Information Figure S2 for high-resolution XPS spectra of C 1s and O 1s). There was a significant decrease in the elemental nitrogen (0.72 at %) and carbon (81.96 at %) compositions in the CIG nanoscaffolds, while there was an apparent increase in the oxygen content (17.32 at %) compared to that in the aniline-functionalized graphene. The observed changes were attributed to the covalent addition of oxygen-rich PMDA (C_10_H_2_O_6_) groups per approximately 55 graphitic carbon atoms. The N 1s spectra of both aniline-functionalized graphene and CIG nanoscaffolds were deconvoluted to give an idea about the covalent interconnection between graphene layers (Figure 2). The high-resolution N 1s XPS spectrum of the aniline-functionalized graphene nanoplatelets displayed a peak at *ca*. 399.78 eV, attributed to *neutral* NH_2_ groups of aniline (Figure 2a).^31^ On the deconvoluted N 1s XPS spectrum, the CIG nanoscaffolds exhibited two contributions having binding energies at *ca*. 400.40 (cont. 80.86%, amidic N atom) and 399.20 (cont. 19.14%) eV, attributable to O=C–N (amide)^32^ and *unreacted* NH_2_ groups,^31^ respectively (Figure 2b). Such a high amidic N atom composition in the CIG nanoscaffolds ensures that the covalent interconnection between graphene nanoplatelets is successfully achieved.

Considering that the average size of any sandwiched NPs can be related to the size of the formed nanogalleries between graphene nanoplatelets, bifunctional linkers can be used to tune the nanogallery size *via* precision covalent chemistry, and one can expect the formation of NPs with particle sizes comparable to that of the graphene layer–layer distance after the chemical functionalization. The simple space-filling model of an ideal CIG nanoscaffold demonstrates an estimated layer–layer distance of ∼2.02 nm (Scheme 1). The present study uses AuNPs as a model system to investigate the size-tuning phenomenon due to their well-known chemistries and widespread use in catalysis^33^ and biomedical applications.^34^ The Turkevich method is known to produce spherical AuNPs, ∼ 10–30 nm in size,^23,24,35^ and it was shown to be effective in decorating reduced graphene oxide surfaces with spherical AuNPs, 14 nm in size.^36^ In our experiments, AuNPs were synthesized using HAuCl_4_/Na_3_Cit (1/6 mol equiv) precursors in an aqueous solution following the Turkevich method^6,23,24^ (Scheme 1). Notably, the 1HAuCl_4_/6Na_3_Cit ratio ensures the formation of approximately 10 nm AuNPs,^23^ and thus the presence of US–AuNPs can be attributed to the well-engineered graphene layer–layer distance.

**Figure 3:**
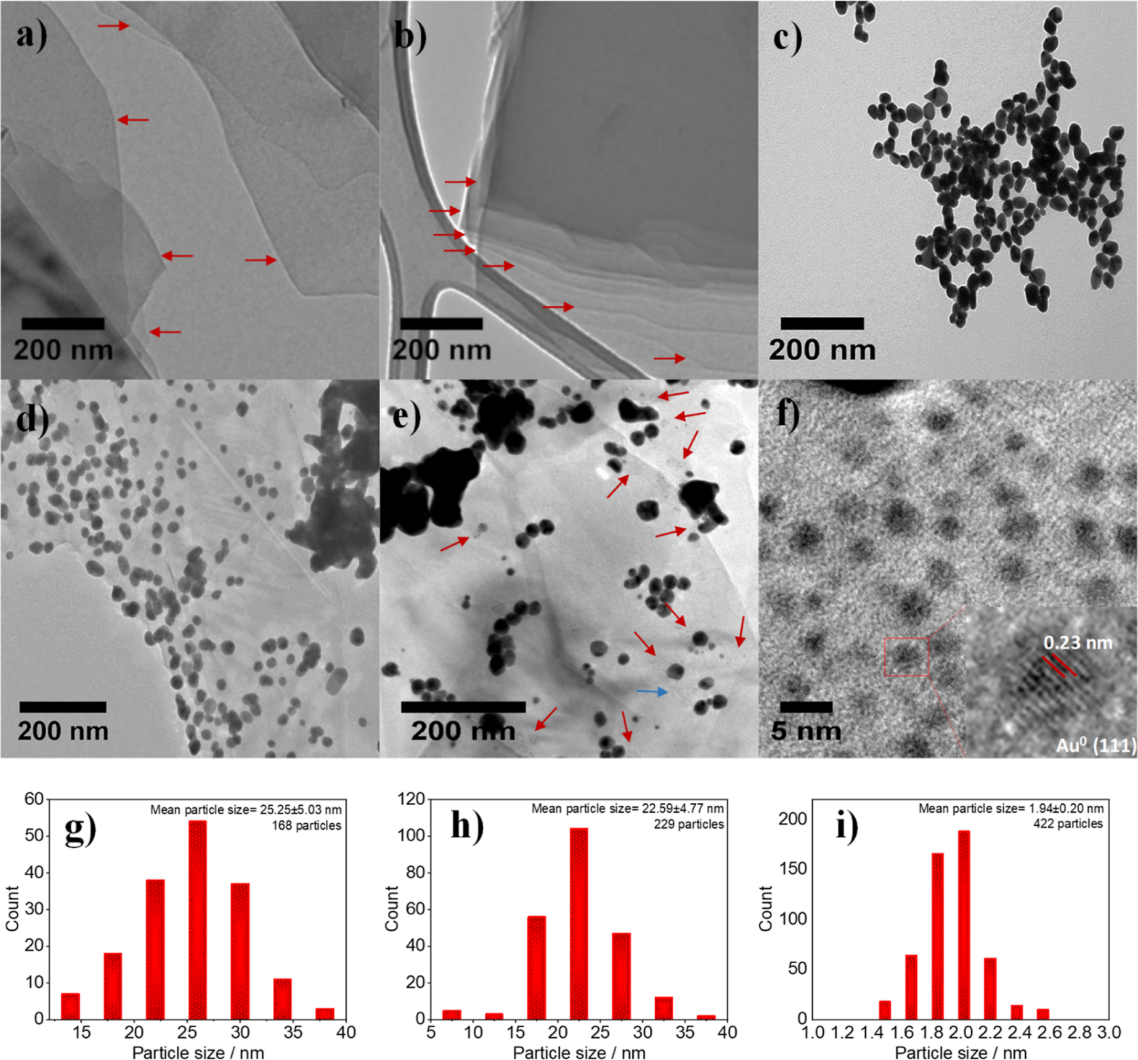
TEM images of (a) exfoliated graphene nanoplatelets, (b) covalently linked few-layer graphene nanoplatelets, (c) AuNPs prepared by the Turkevich method with no graphene nanoplatelets, and (d) AuNP/aniline-functionalized graphene hybrid structure. (e) TEM image of the US–AuNP/CIG nanoscaffold hybrid structure. Red and blue arrows show the regions in which US–AuNPs are located. (f) HRTEM image of AuNPs generated (the region shown by the blue arrow in (e) between the covalently linked graphene nanoplatelets. The inset shows a lattice spacing of 0.23 nm, characteristic of Au^0^ (111), confirming AuNP formation. Size distribution of AuNPs prepared (g) with no graphene and in the presence of (h) aniline-functionalized graphene and (i) CIG nanoscaffolds.

Analysis of the graphene dispersion by transmission electron microscopy (TEM) shows randomly stacked few-layer graphene nanoplatelets (Figure 3a), which well agrees with the previous literature.^37^ In contrast, the TEM image of the CIG nanoscaffolds displays more organized layer arrangements (Figure 3b), attributed to the covalent interconnection. The AuNPs produced without graphitic support show a mean particle size of 25.25 ± 5.03 nm (Figure 3c,g). On the other hand, the AuNPs prepared in the presence of the aniline-functionalized graphene show a slightly smaller mean particle size (22.59 ± 4.77 nm) (Figure 3d,h), which may be related to the stabilization of AuNPs by NH_2_ groups on aniline. Furthermore, the smallest particle size analyzed by high-resolution (HR)TEM is about 7–8 nm (Figure 3d and Supporting Information Figure S3 for additional TEM images). However, a few large aggregates (≥200 nm) are also present on the graphene surface (Figure 3d). In contrast, the HRTEM image of AuNPs prepared in the presence of the CIG nanoscaffolds, with a preadjusted layer–layer distance of ∼2.02 nm, displays islands with homogeneously distributed US–AuNPs (labeled by arrows) (Figure 3e). Closer inspections of the HRTEM image of those islands (Figure 3f,i and inset) show US–AuNPs with a mean particle size of 1.99 ± 0.20 nm and a lattice spacing of 0.23 nm, characteristic of Au^0^ (111).^19^ The experimentally derived mean particle size of US–AuNPs (1.99 ± 0.20) is in very close agreement with the estimated layer–layer distance of the CIG nanoscaffolds (∼2.02 nm). In addition, there are AuNPs (∼10–25 nm) and some large aggregates (≥200 nm) on the CIG surface, which are comparable to the results obtained for the aniline-functionalized graphene. However, no US–AuNP is observed in HRTEM images of the aniline-functionalized graphene, suggesting that they can be produced only when the layer–layer distance is finely tuned. Overall, HRTEM results underscore that the fine-tuning of AuNPs between graphene nanoplatelets can be achieved *via* precise covalent chemistry.

Further evidence of the finely tuned graphene layer–layer distance and the accompanying US–AuNP formation comes from the lattice spacing distance of the CIG nanoscaffolds analyzed by HRTEM. Theoretically, the distance between two graphene layers is ∼0.335 nm, and the thickness of a single layer of graphene measured by atomic force microscopy (AFM) varies from 0.4 to 1.0 nm.^38,39^ In addition, the spacing between graphene layers can be altered *via* intercalation,^20^ or covalent cross-linking chemistries,^40,41^ and the covalently interconnected graphene architectures can be characterized by HRTEM^41,42^ using lateral distances.

**Figure 4:**
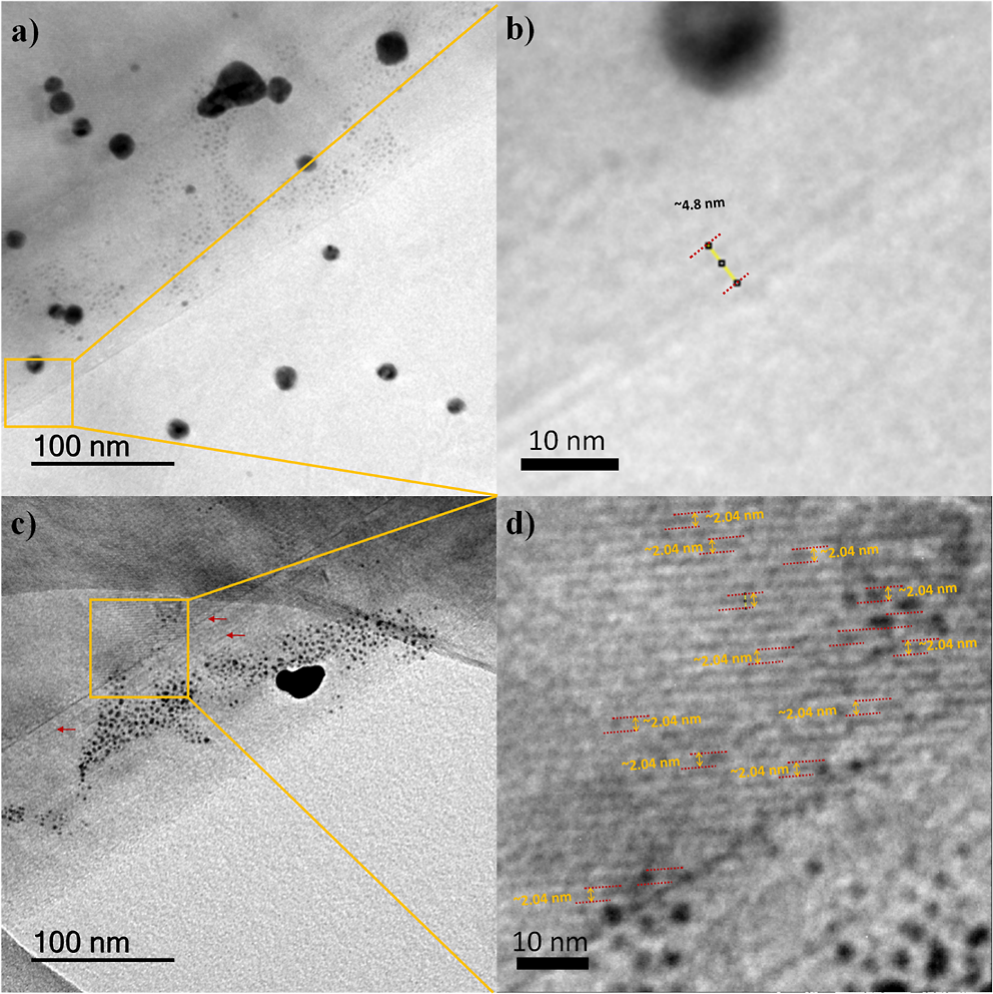
(a) TEM image of the US–AuNP/CIG nanoscaffold showing a three-layer covalently linked graphene architecture, (b) enlarged region TEM image of the US–AuNP/CIG nanoscaffold showing the thickness (∼4.8 nm) of the three-layer graphene architecture, (c) TEM image of the US–AuNP-decorated CIG nanoscaffold hybrid structure showing the lattice spacing of the covalently linked graphene architecture, and (d) enlarged region TEM image of the US–AuNP/CIG nanoscaffold with a lattice fringe of ∼2.04 nm.

Agreeing with Raman spectroscopy, close inspection of the additional HRTEM image of CIG nanoscaffolds shows a three-layer graphene architecture decorated with US–AuNPs (Figure 4a,b).^42^ Assuming that three-layer graphene has a thickness of ∼1.0 nm (by AFM),^38^ the measured distance of ∼4.8 nm well agrees with three-layer graphene, which is covalently interconnected by two PMDA bridges. Another complementary HRTEM image of the CIG nanoscaffold displays US–AuNP-decorated surfaces, and the graphene layers are visibly separated (Figure 4c,d). The measured lattice spacing distance of the CIG nanoscaffolds is ∼2.04 nm, undoubtedly indicating the covalent interconnection *via* bifunctional PMDA linkers. Furthermore, the experimentally acquired layer–layer distance of the CIG nanoscaffolds agrees well with the estimated layer–layer distance of ∼2.02 nm.

**Figure 5:**
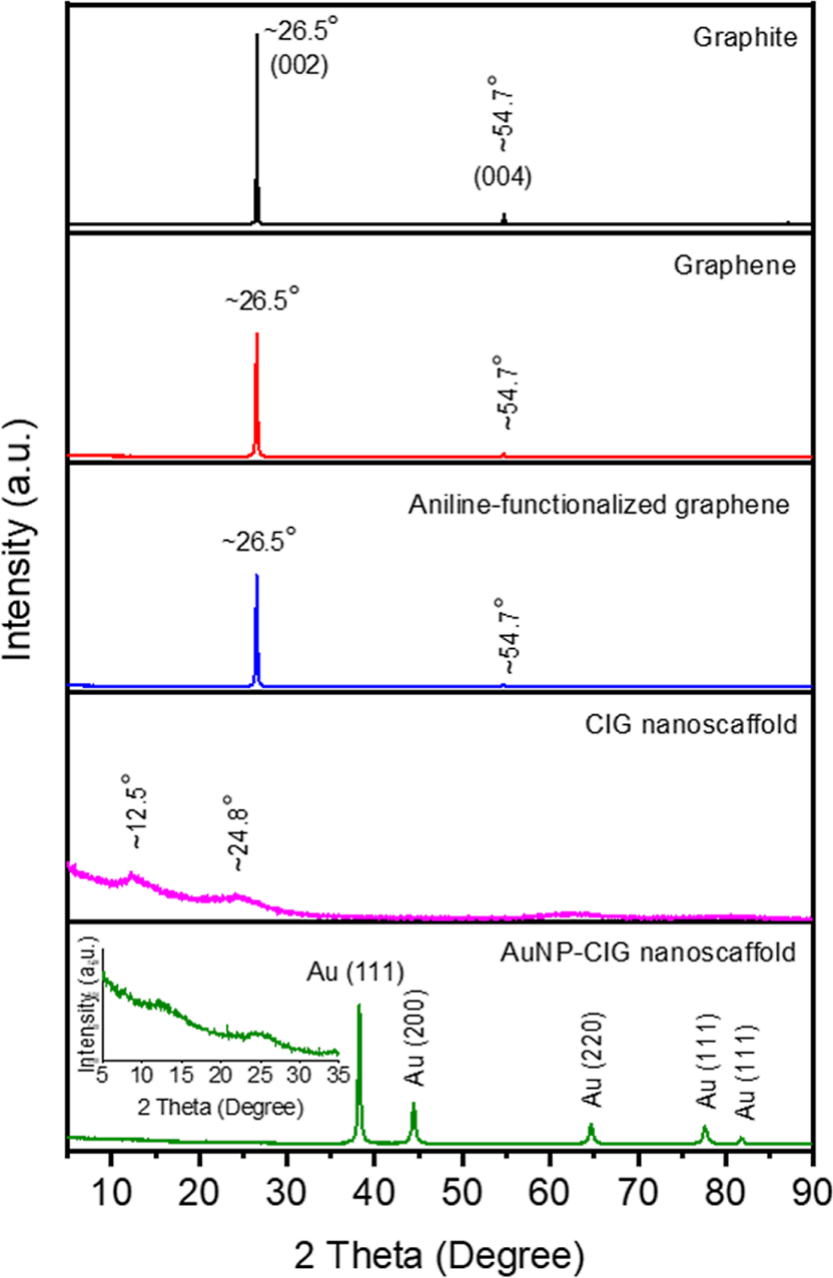
XRD patterns of natural graphite, exfoliated graphite, aniline-functionalized graphene, CIG nanoscaffold, and AuNP-CIG nanoscaffold. The inset shows the enlarged area (5–35°).

Complementary X-ray diffraction data of natural graphite, exfoliated graphite, aniline-functionalized graphene, CIG nanoscaffold, and AuNP-CIG nanoscaffold are presented in Figure 5. For as-received graphite, the crystalline planes (002) and (004) were obtained at *ca*. 26.5 and 54.7°, respectively.^43^ No significant change in the position of (002) and (004) reflections was observed after exfoliation and aniline functionalization of graphite flakes; however, the intensity of the (002) peak significantly decreased, attributable to less-layered nanosheets, compared to that of the as-received graphite flakes. The PMDA-bridged CIG nanoscaffold displayed no (002) and (004) reflections; in contrast, two new peaks appeared at *ca.* 24.8 and 12.5°, attributed to covalently bridged graphene layers with PMDA spacers, which correlates well with an increase in the layer–layer distance between graphene nanosheets. Such a low-angle shifting phenomenon compared to the (002) reflection at ∼26.5° was also observed in previous studies, which used rigid linkers (*e.g.*, phenyl, biphenyl, and *p*-terphenyl) to prepare covalently connected graphenes.^41^ After AuNP incorporation into the CIG nanoscaffold, there was no change in the peaks at *ca.* 24.8° and 12.5°; however, AuNP-related five peaks at *ca*. 38.2, 44.4, 64.6, 77.6, and 81.7° were attributed to standard Bragg reflections of (111), (200), (220), (311), and (222) for the face-centered cubic crystal structure, respectively.^44^

## Conclusions

In summary, a hybrid US–AuNP (mean particle size of 1.94 ± 0.20 nm)/graphene nanoplatelet sandwich was fabricated using the CIG nanoscaffolds with a pretuned graphitic layer–layer distance of ∼2.04 nm. The precision covalent chemistry strategy to prepare sandwich structures significantly differs from the sequential layer-by-layer stacking^17^ and sacrificed template^18^ techniques reported in the literature. The linker molecule, PMDA, principally predetermines the layer–layer distance of graphene nanoplatelets and the size of the sandwiched nanoparticles. When ease of access to commercially available dianhydrides (*e.g.*, ethylenediaminetetraacetic dianhydride, benzophenone-3,3′,4,4′-tetracarboxylic dianhydride, and perylene-3,4,9,10-tetracarboxylic dianhydride) and other bifunctional cross-linking agents was combined with well-practiced carbon nanomaterials covalent surface modification approaches,^45,46^ an acceptable size control on sandwiched nanoparticles in between graphene nanosheets can be realized for several applications from energy storage materials^47−49^ to electromagnetic shielding^50^ and membranes.^51^ In addition to 2D graphene nanoplatelets, given the available covalent surface modification techniques, a library of 2D materials such as MoS_2_,^52,53^ h-BN,^54^ TiS_2_,^55^ MoSe_2_,^55^ and Bi_2_Te_3_^55^ can be processed similarly to prepare hierarchical nanoscaffolds of other 2D-materials. This elegant approach may provide precise size control while producing sandwiched metal/metal oxide nanoparticles between 2D-layered nanostructures, whose surfaces are prone to covalent chemistries.

## Supporting Information Available

UV–vis spectrum of graphene dispersion, C 1s and O 1s XPS spectra of the functionalized graphene, and additional HRTEM images of the AuNP/graphene nanoplatelets (PDF)
