# Supplementary material for: Precision Covalent Chemistry for Fine-Size Tuning of Sandwiched Nanoparticles between Graphene Nanoplatelets
Source: ACS Omega. 2023 Oct 27;8(44):41273–81. doi: 10.1021/acsomega.3c04727 (PMC10633857; doi:10.1021/acsomega.3c04727)
Supplement: Supplementary file 1 — ao3c04727_si_001.pdf [file ao3c04727_si_001.pdf]

# Supporting Information

## Precision covalent chemistry for fine-size tuning of sandwiched nanoparticles between graphene nanoplatelets

Mustafa K Bayazit[1,2,3]

[1] Sabanci University Nanotechnology Research and Application Center, Tuzla Istanbul, 34956, Turkey
[2] Faculty of Engineering and Natural Science, Sabanci University, 34956 Istanbul, Turkey
[3] Department of Chemical Engineering, University College London, Torrington Place, London WC1E 7JE, UK

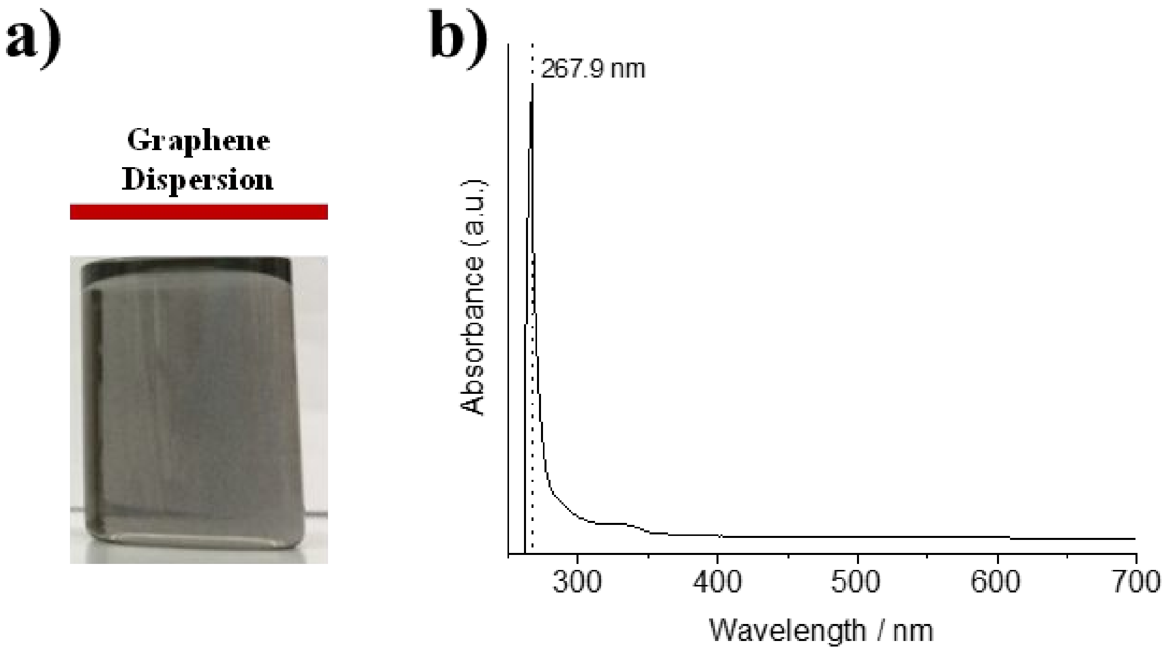


**Figure S1 a)** Photograph of exfoliated graphene prepared in DMF **b)** UV-vis spectrum of graphene dispersion in DMF.

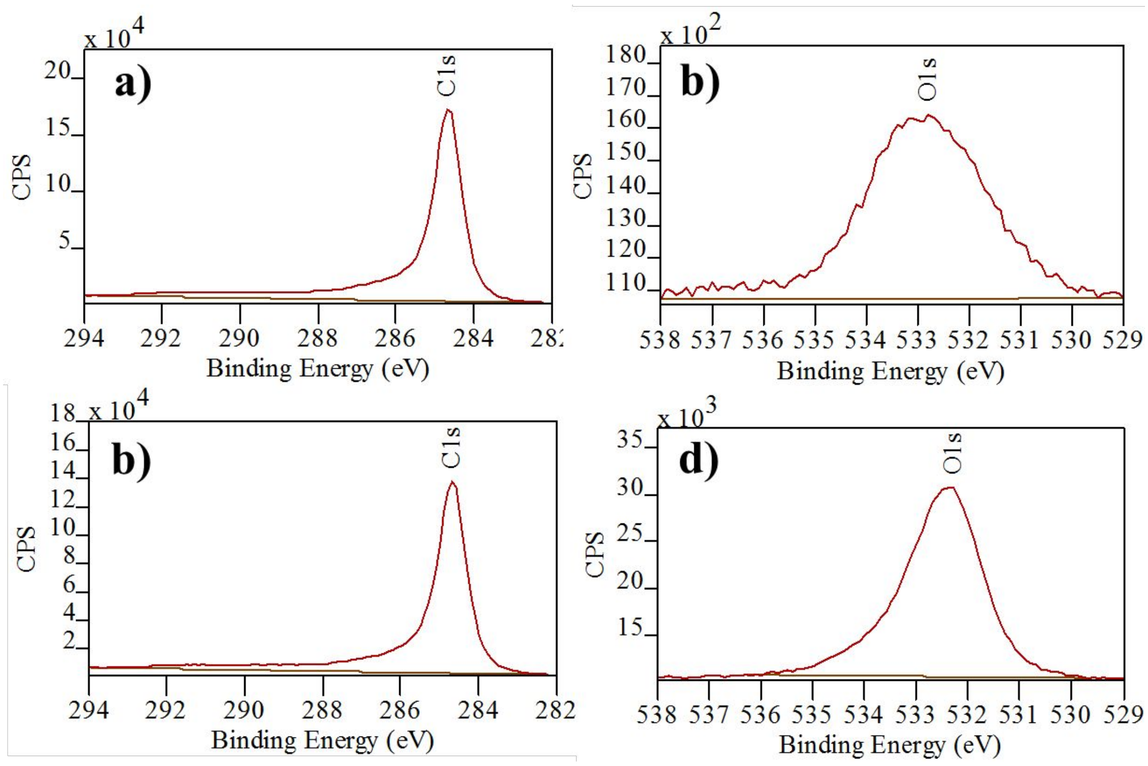


**Figure S2 a)** C 1s and **b)** O 1s XPS spectra of the aniline functionalized graphene. **c)** C 1s and **d)** O 1s XPS spectra of the covalently linked graphene nanoplatelets.

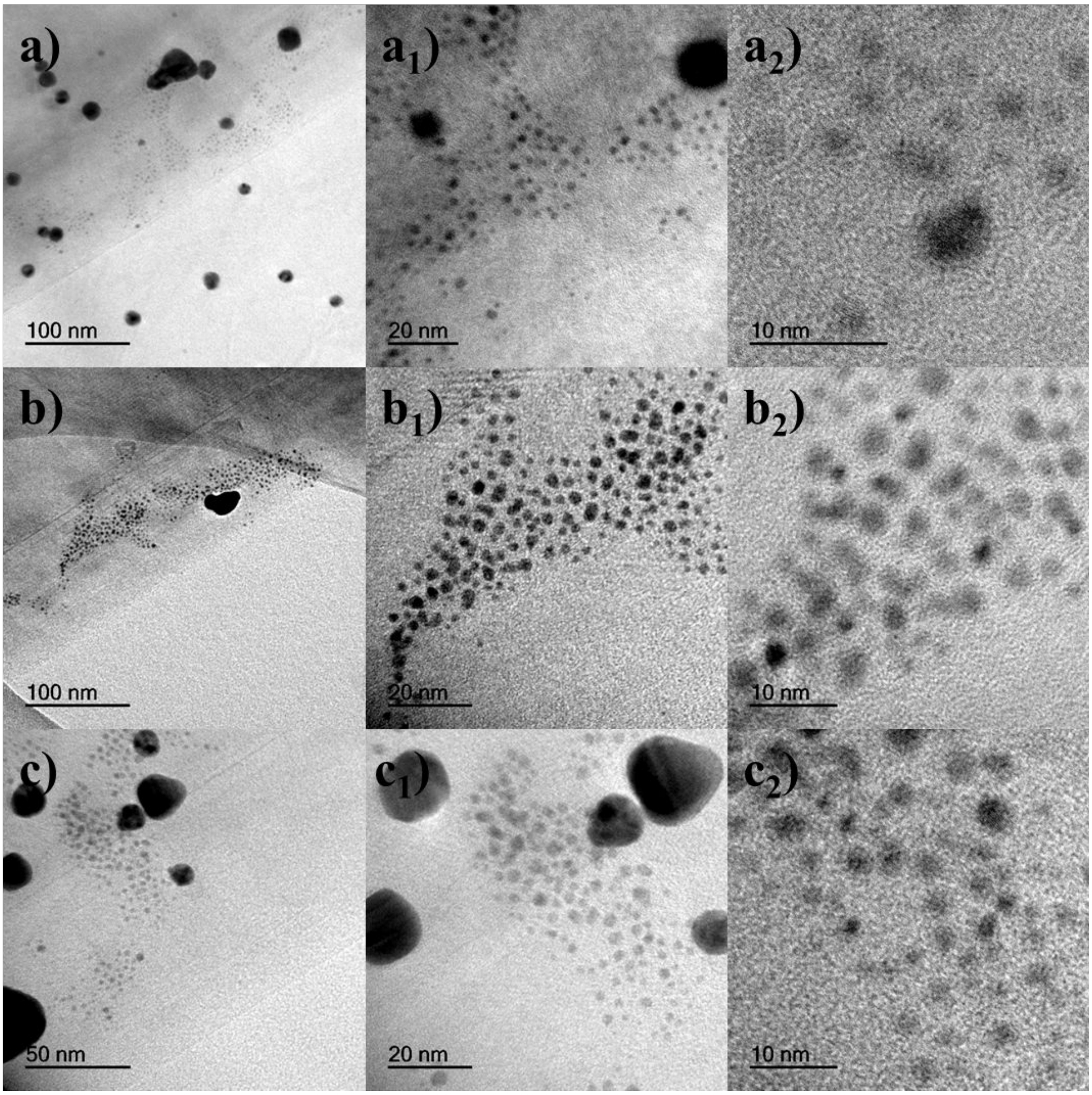
a)
100 nm
a1)
20 nm
a2)
10 nm
b)
100 nm
b1)
20 nm
b2)
10 nm
c)
50 nm
c1)
20 nm
c2)
10 nm

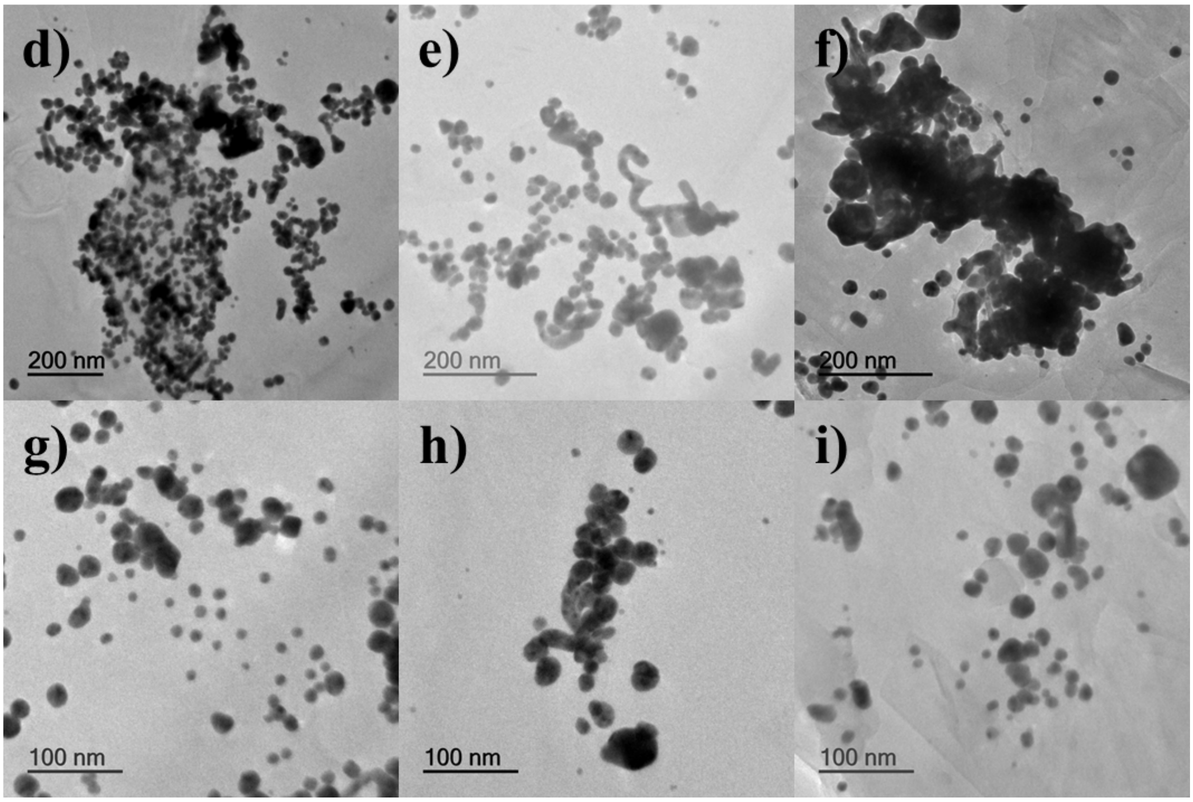


**Figure S3** Additional HRTEM images (**a-c)** and their enlarged regions (**a**$_{1-2}$, **b**$_{1-2}$ and **c**$_{1-2}$**)** of the AuNP/covalently linked graphene nanoplatelets. **d-i)** Additional HRTEM images of the AuNP/aniline functionalized graphene hybrids. No ultra-small gold nanoparticle (~2 nm) is observed in TEM images of AuNP/aniline functionalized graphene hybrids, indicative of random Au-NP formation.
